# Mining the Drilosphere: Bacterial Communities and Denitrifier Abundance in a No-Till Wheat Cropping System

**DOI:** 10.3389/fmicb.2019.01339

**Published:** 2019-06-26

**Authors:** Daniel C. Schlatter, Catherine L. Reardon, Jodi Johnson-Maynard, Erin Brooks, Kendall Kahl, Jessica Norby, David Huggins, Timothy C. Paulitz

**Affiliations:** ^1^Wheat Health, Genetics and Quality Research Unit, Agricultural Research Service, United States Department of Agriculture, Pullman, WA, United States; ^2^Soil and Water Conservation Research Unit, Agricultural Research Service, United States Department of Agriculture, Adams, OR, United States; ^3^Department of Soil and Water Systems, University of Idaho, Moscow, ID, United States; ^4^Northwest Sustainable Agroecosystems Research Unit, Agricultural Research Service, United States Department of Agriculture, Pullman, WA, United States

**Keywords:** earthworms, soil, microbiome, next-generation sequencing, direct seed, Pacific Northwest

## Abstract

Earthworms play important roles in no-till cropping systems by redistributing crop residue to lower soil horizons, providing macropores for root growth, increasing water infiltration, enhancing soil quality and organic matter, and stimulating nitrogen cycling. The soil impacted by earthworm activity, including burrows, casts, and middens, is termed the drilosphere. The objective of this study was to determine the effect of earthworms on soil microbial community composition in the drilosphere at different landscape slope positions. Soil cores (50 cm depth) were extracted from three landscape locations (top, middle, and bottom slope positions) on a sloping aspect of a no-till wheat farm. Soil was sampled at the bottom of the soil core from inside multiple earthworm (*Lumbricus terrestris*) channels (drilosphere) and from adjacent bulk soil. Bacterial communities were characterized for 16S rRNA gene diversity using high-throughput sequencing and functional denitrifier gene abundance (*nirK, nirS*, and *nosZ*) by quantitative PCR. Bacterial communities were structured primarily by the landscape slope position of the soil core followed by source (bulk versus drilosphere soil), with a significant interaction between core position and source. The families AKIW874, Chitinophagaceae, and Comamonadaceae and the genera *Amycolatopsis, Caulobacter, Nocardioides*, and *Variovorax* were more abundant in the drilosphere compared to the bulk soil. Most of the individual bacterial taxa enriched in the drilosphere versus bulk soil were members of Actinobacteria, including Micrococcales, Gaiellaceae, Solirubrobacterales, and *Mycobacterium*. In general, the greatest differences in communities were observed in comparisons of the top and bottom slope positions in which the bottom slope communities had significantly greater richness, diversity, and denitrifier abundance than the top slope position. Populations of denitrifiers (i.e., ratio of *nirK*+*nirS* to 16S rRNA) were more abundant in earthworm-impacted soils and there was a significant impact of *L. terrestris* on soil community composition which was observed only in the top landscape position. There were significant correlations between the abundance of *nirK* and *nirS* and taxa within Proteobacteria, Acidobacteria, Actinobacteria, Verrucomicrobia, and Chloroflexi, suggesting a broad diversity of denitrifying bacteria. Earthworms influence the soil microbial communities, but the impact depends on the slope location in a variable landscape, which likely reflects different soil characteristics.

## Introduction

As ecosystem engineers, earthworms play a major role in the formation of soil and have profound effects on the physical, chemical, and biological properties of the below-ground environment (Lavelle et al., 2016). Earthworms are important in agricultural systems for litter decomposition (Cortez, 1998), microaggregate formation (Pulleman et al., 2005), penetration of roots through compacted soil (Edwards and Lofty, 1980), and water infiltration (Willoughby et al., 1997). Additionally, positive effects of earthworms on crop productivity have been observed for both crop yield and aboveground biomass, with the plant benefit attributed to the release of nitrogen from organic matter and crop residues (van Groenigen et al., 2014). The volume of soil impacted by earthworms, termed drilosphere, includes casts, middens, burrows, and burrow linings, and influences from physical features of earthworms (body surface, gut, and internal features) and secretions (e.g., mucus, sloughed off cells) (Brown et al., 2000).

As a result, the drilosphere is enriched in labile carbon promoting microbial “hotspots” with increased microbial and macrofaunal biomass (Tiunov et al., 2001; Don et al., 2008; Stromberger et al., 2012), as well as soil enzyme activity for organic matter breakdown compared to the bulk soil (Görres et al., 2001; Don et al., 2008; Beloqui et al., 2010; Hoang et al., 2016a,b). Using labeled carbon (C) and nitrogen (N), Andriuzzi et al. (2013) calculated the dimensions of the zone of influence to be greater than 2 mm, possibly 4–8 mm in soil surrounding earthworm burrows.

In minimum disturbance systems, deep-burrowing anecic earthworms are important in burying surface residue in the soil profile for decomposition. *Lumbricus terrestris*, colloquially known as a nightcrawler, is an introduced species that is now commonly found in natural and agricultural soils throughout North America. These anecic earthworms create deep vertical burrows in soil that can increase water infiltration and O_2_ levels in soils (Willoughby et al., 1997), and provide preferential pathways for growing plant roots (Edwards and Lofty, 1980; Cameron et al., 2014). The burrows can remain in soil as stable structures lasting up to 7 years, where they are spatiotemporally stable reservoirs of soil resources (Potvin and Lilleskov, 2017). Earthworms translocate organic matter into these burrows by the physical movement of large amounts of plant residue (Curry and Schmidt, 2007) and by the deposition of mucus and casts. Earthworm activity results in significant changes in soil properties, such as greater amounts of microbial biomass C and N, mineralizable C, and nitrate compared to undisturbed soil (Burtelow et al., 1998). In addition, earthworm-impacted soils also exhibit greater denitrification activity than bulk soils (Burtelow et al., 1998).

The earthworm gut is anoxic, high in organic C and inorganic N, and host to abundant populations of denitrifying bacteria (Horn et al., 2003). Denitrifying population densities in the earthworm gut can far exceed that of the bulk soil. For example, the abundance of cultivable denitrifiers in the *Lumbricus rubellus* gut are 256-times greater than in bulk soil (Karsten and Drake, 1997). The earthworm gut emits N_2_O and N_2_, both of which are attributed to the gut microbial communities (Ihssen et al., 2003; Horn et al., 2006; Nebert et al., 2011; Chen and Whalen, 2016).

It is hypothesized that the denitrifying populations are derived from soil rather than being gut-specific, and that the microenvironment of the gut promotes N_2_O-production (Horn et al., 2003). Similarly, earthworm guts and casts have greater capacities for denitrification than the surrounding soil (Svensson et al., 1986; Matthies et al., 1999). However, little is known regarding the microbial community composition and structure of the earthworm drilosphere, which is also enriched in microbial biomass and activity (respiration) compared to non-impacted or nearby bulk soil (Tiunov and Scheu, 1999; Tiunov and Kuznetsova, 2000).

With some exceptions, microbial communities in the drilosphere are poorly characterized. Studies in the late 1990s with culturable bacteria showed high levels of *Pseudomonas* and siderophore-producing bacteria (Devliegher and Verstraete, 1997) and *Bacillus* (Biswas et al., 2014). Byzov et al. (2009) cultured bacteria and fungi from soil and casts and identified them by sequencing the bacterial 16S rRNA and fungal 28S rRNA (D1/D2 domain) gene sequences. They identified the families Aeromonadaceae, Comamonadaceae, Enterobacteriaceae, Flavobacteriaceae, Moraxellaceae, Pseudomonadaceae, and Sphingobacteriaceae. Using phospholipid fatty acid analysis (PLFA), Stromberger et al. (2012) found distinct microbial and faunal communities in the *L. terrestris* drilospheres, with greater abundance of microbes, microfauna including protozoa, nematodes, and mesofauna (e.g., Collembola), in the drilosphere compared to nearby bulk soil. Similarly, Banfield et al. (2017) used PLFA to show higher levels of microbes in both the drilosphere and biopores. Using Single-Strand Confirmation Polymorphisms (SSCP) and sequence analysis of 16S rRNA clone libraries, Nechitaylo et al. (2010) showed that *L. terrestris* contained representatives of classes Flavobacteria and Sphingobacteria (Bacteroidetes) and *Pseudomonas* spp. using Automated Ribosomal Intergenic Spacer Analysis (ARISA), Thakuria et al. (2010) demonstrated that the bacteria in guts of different ecological groups (anecic *L. terrestris* and *L. friendi*, the endogeic *Aporrectodea caliginosa* and *A. longa*) were distinct, and that habitat was a greater determinant than earthworm species.

In this study, we evaluated effects of earthworms on the soil microbial communities focusing on differences between bulk and drilosphere soil. The study was conducted to evaluate whether bacterial communities of drilosphere soil differed from those of bulk soil.

In the Palouse region of eastern Washington state, high wheat yields (average yield 4.43 tons ha^-1^ in Whitman county; NASS Quick Stats) leave a tremendous amount of wheat residue on the soil surface in direct-seed systems (around 3500 kg C ha^-1^ yr^-1^) (Huggins et al., 2014). The combination of high residue inputs and reduced or no tillage promotes greater earthworm densities due to the provision of a large food base and reduced disturbance (Peigné et al., 2009; Rothwell et al., 2011). Few studies, however, have used high-throughput DNA sequencing (HTS) to examine microbial communities associated with *L. terrestris* in the drilosphere. The objective of this research was to use HTS to characterize the bacterial communities of the drilosphere of *L. terrestris* under a direct-seed system, and compare them to the adjacent bulk soil. We hypothesized that the drilosphere contains a more rich and diverse community than bulk soil and is enriched in denitrifying populations and taxa capable of breaking down crop residues.

## Materials and Methods

### Site Description

The study was conducted in the dryland, temperate Palouse region of the inland Pacific Northwest which is noted for its topographically sloping agricultural landscape. Samples were collected from three slope positions (top-slope, mid-slope, and bottom-slope) at the R.J. Cook Agronomy Farm (CAF), a USDA Long-Term Agroecosystem Research (LTAR) site in WA, United States (46° 46′ 44″ N, 117° 05′ 19″ W). The CAF has been managed in a 3-year winter wheat-spring grain-pulse crop rotation following no-tillage practices since 1998. Historically water and tillage-induced erosion across the region have dramatically altered the distribution of soils within these landscapes (USDA, 1978). Upper eroded locations (top-slopes) often have less topsoil and produce lower yields than deposition regions (Fiez et al., 1995). However, the soil was classified by USDA-ARS soil scientists a Palouse silt loam (Fine-silty, mixed, superactive, mesic, Pachi, Ultic, Haploxerolls) having a well-drained mollic epipedon from the surface to 61 cm over a thick cambic horizon from 61 to 150 cm. The bottom-slope position was a Thatuna silt loam, fine-silty, mixed, superactive, mesic Oxyaquic Argizerolls which has a mollic epipedon zone from the surface to a depth of 69 cm, followed by an albic horizon from 69 to 94 cm, and a dense argillic horizon from 94 to 163 cm. The mid-slope was a Palouse silt loam soil. In comparison to field averages, ridge locations characteristic of the top-slope core position produce 20% less crop yield; whereas, the north facing slope of the bottom- and mid-slope core positions normally yield 20% greater than the field average (Huggins et al., 2014). Soil horizon descriptions for each of the landscape positions are provided in Supplementary Table 1.

### Soil Sampling

**FIGURE 1:**
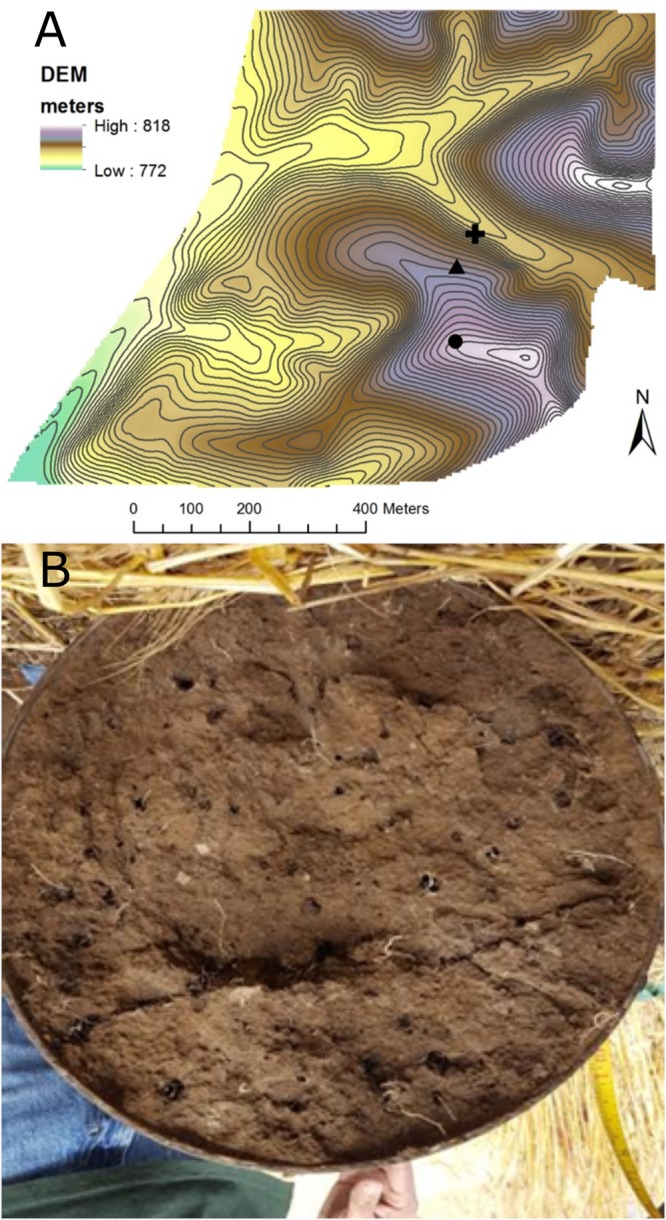
**(A)** Map showing the locations of the top-slope (circle), mid-slope (triangle), and bottom-slope (plus sign) core positions within the Cook Agronomy Farm. Contour intervals are 1 m. **(B)** Example of earthworm burrows found in the 25 cm (10 inch) diameter extracted soil cores (viewed from the bottom at a depth of 50 cm).

Large, undisturbed soil cores were collected October 6, 2016 from the top-slope (811.6 m elevation), mid-slope (801.6 m), and bottom-slope (791.6 m) at CAF (Figure 1A). Steel cylinders (25-cm inside diameter by 50 cm long) were driven vertically into the soil using an electric powered post driving machine. The soil surrounding the core was removed manually by digging, and the cylinder retaining the soil core carefully lifted and transported to the lab. Many earthworm burrows were visible at the bottom (50-cm depth) of each core (Figure 1). Earthworm burrows (*n* = 6 per core) were sampled from the core bottoms by scraping the inside of an individual channel with a sterilized powder scoop. We could not distinguish active from abandoned earthworm burrows; however, sampled burrows were large, continuous and lacked clear signs of root growth of substantial enough size to limit earthworm use of the burrows. Bulk soil was sampled (*n* = 4) from arbitrary points on core bottoms at a distance of at least 5 mm away from the earthworm channels (Figure 1B). Bulk density was measured at the collection site from replicate cores (5 cm diameter × 5 cm length) extracted from the side walls of the hole at three depths (0–5 cm, 20–25 cm, 35–40 cm) (Supplementary Table 2). While earthworms were not directly sampled, middens were present throughout the CAF field, with an average value of 11 ± 6 middens m^-2^ measured on the same slope positions and aspects within 175 m of where the drilosphere cores were taken (data not shown). In a recent survey of agricultural fields in the inland Pacific Northwest, which included the CAF, *L. terrestris* was the only anecic, midden-producing species sampled from fields located in the annual cropping region (Walsh and Johnson-Maynard, 2016).

### DNA Extraction and Amplicon Sequencing

DNA was extracted from 0.25 g soil using the PowerSoil DNA extraction kit (Qiagen/MoBio, Carlsbad, CA, United States) according to the manufacturer’s instructions with bead-beating performed on a FastPrep system using the “soil” setting. The amount of DNA extracted from the burrows was similar across the landscape position (average concentration of 5.7 ng μl^-1^) and the amount of DNA extracted in bulk soil varied across slope position with 1.4 ng μl^-1^ from the top slope, 11.3 ng μl^-1^ from midslope and 16.9 ng μl^-1^ from the bottom slope. DNA was normalized, amplified using 27F (Edwards et al., 1989) and 518R (Øvreås et al., 1997) primers targeting the V1–V3 region of the 16S rRNA gene, barcoded, and sequenced on the Illumina MiSeq platform (Version 3 chemistry, 2 × 300 paired-end reads) according to established protocols (Gohl et al., 2016).

### Quantitative PCR

Bacteria and denitrifying populations were quantified by qPCR based on the abundance of genes encoding the bacterial 16S rRNA, copper nitrite reductase (*nirK*), *cd*_1_ nitrite reductase (*nirS*), and nitrous oxide reductase (*nosZ*) (Supplementary Table 3). DNA extracts were diluted 1:20 and tested for the presence of PCR inhibitors according to published protocols prior to quantification (Reardon et al., 2014). None of the extracts showed signs of PCR inhibition and the C_q_ (quantification cycle) of reactions with soil DNA varied less than 0.3 C_q_ from the soil DNA-free control. Quantification was performed in 10 μl reactions with 0.8× Power SYBR Green Master Mix (Life Technologies, Grand Island, NY), 0.1 μg μl^-1^ bovine serum albumin (Roche Applied Science, Indianapolis, IN, United States), forward and reverse primer (Supplementary Table 3), and 1 μl DNA diluted 1:20. Standard curves were generated by 10-fold dilution of plasmid containing the gene targets for a range of 5–6 orders of magnitude. Thermocycling conditions are presented in the Supplementary Table 4. Fluorescence was measured at the end of each complete cycle and the amplification was followed by a final melt curve. All protocols were specifically optimized for the SYBR reagent and instrument.

### Data Processing

Read preparation and operational taxonomic unit (OTU) clustering were performed according to the UPARSE pipeline (Edgar, 2013) as described in the Supplementary Table 4.

Taxonomy was assigned to OTU representative sequences (centroids) with the RDP Naïve Bayesian Classifier (Wang et al., 2007) using the Greengenes 13_8 reference database and an 80% confidence threshold. OTUs were filtered to remove any OTUs that could not be classified to the kingdom Bacteria or those classified as mitochondria or chloroplasts using QIIME scripts (v1.9.1) (Caporaso et al., 2010). OTUs with a total sequence count of <10 were removed to reduce poor quality OTUs and the OTU table was rarefied to 13,000 sequences/sample prior to analysis. Unrarefied OTU tables were retained for differential abundance analysis with DESeq2 (v1.12.4) (Love et al., 2014).

### Data Analysis

Non-metric multidimensional scaling (NMDS) and PERMANOVA were performed using Bray-Curtis dissimilarity among samples to assess the significance of the landscape position of the core and sample source (bulk soil versus earthworm drilosphere) on bacterial community structure using the metaMDS and adonis functions of the vegan package v2.4.1 (Oksanen et al., 2016) in R (R Core Team, 2016). OTU richness and diversity metrics (Shannon’s [H’] and inverse Simpson’s [1/D]) were estimated from rarefied OTU tables and tested using ANOVA. Bacterial families and genera that differed in relative abundance between bulk soil and earthworm channels were evaluated with Kruskal–Wallis tests on abundant groups (>0.5% of sequences), followed by correction for false discovery using the Benjamini-Hochberg procedure. DESeq2 was used to identify differentially abundant OTUs (DAotus) between bulk or drilosphere soil. Briefly, unrarefied OTU tables were filtered to contain only OTUs with normalized counts >20 and present in three or more samples. DAotus were evaluated after accounting for core identity using the model: ∼Core Position × Soil Source. Contrasts were performed between bulk and channel soil for each core and OTUs with adjusted *p*-values of <0.001 and log2-fold changes >2 were considered to be differentially abundant. Relationships between abundant OTUs (>100 sequences in rarefied data) and copy numbers of *nirK* and *nirS* genes were evaluated using Spearman correlations. Finally, the abundance of *nirK* and *nirS* genes and their ratios with 16S rRNA gene abundance were compared among landscape positions and between bulk and drilosphere soil with ANOVA and nested ANOVA, respectively.

## Results

**FIGURE 2:**
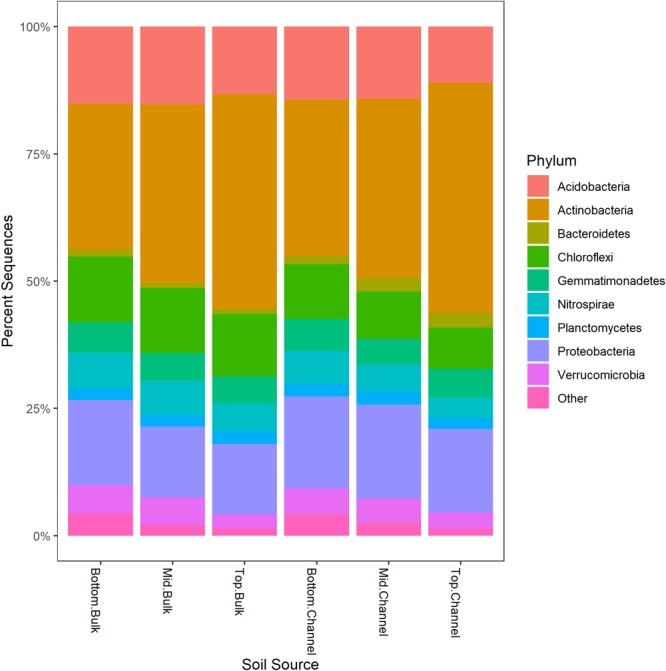
Relative abundance [Log_2_(1+x)-transformed rarefied sequence counts] of bacterial phyla among slope positions (top-, mid-, bottom-slope) and soil sources (bulk, channel). Top, mid, and bottom refer to slope positions.

**FIGURE 3:**
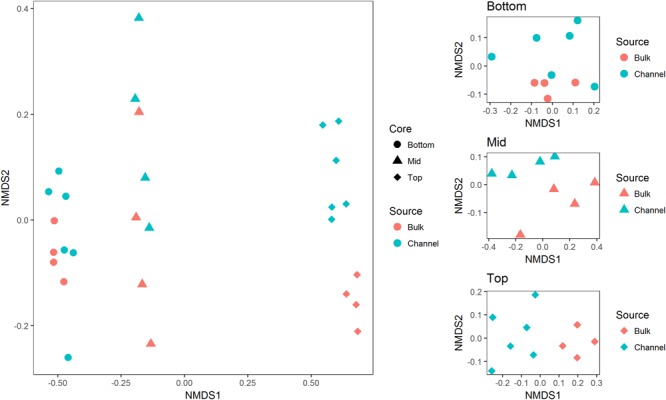
Non-metric multidimensional scaling plots of bacterial communities among (left; Stress = 0.044), and within individual soil cores (right; Stress = 0.09, 0.02, and 0.06 for bottom-, mid-, and top-slope samples, respectively). Top, mid, and bottom refer to slope positions.

Sequence processing resulted in a total of 749,959 sequences belonging to 5,509 bacterial OTUs among 30 samples (364,000 sequences belonging to 4,322 OTUs after rarefaction). Bacterial communities from both bulk soil and earthworm channels were dominated by Actinobacteria (29–42% and 30–45%, respectively), Proteobacteria (14–17% and 16–18%, respectively), and Acidobacteria (13–15% and 11–14%, respectively), with other common phyla including Chloroflexi, Firmicutes, Nitrospirae, Gemmatimonadetes, and Verrucomicrobia (Figure 2). Overall, bacterial communities were affected primarily by the landscape slope position of the soil core (Figure 3; adonis *r*^2^ = 0.498, *p* = 0.001), followed by source (bulk versus drilosphere soil; Figure 3; adonis *r*^2^ = 0.075, *p* = 0.001) with a significant interaction between core slope position and source (Core Position × Source adonis *r*^2^ = 0.058, *p* = 0.04). However, within individual soil cores, soil source explained a significant amount of variation in bacterial community structure only in the top-slope core (adonis *r*^2^ = 0.39, *p* = 0.002; Figure 3). Thus, within-field variation in microbial community composition was a larger factor in determining overall bacterial community structure than earthworm-soil interactions. However, the significance of the drilosphere effect for bacterial communities in earthworm macropores depended on landscape slope position (Figure 3).

**FIGURE 4:**
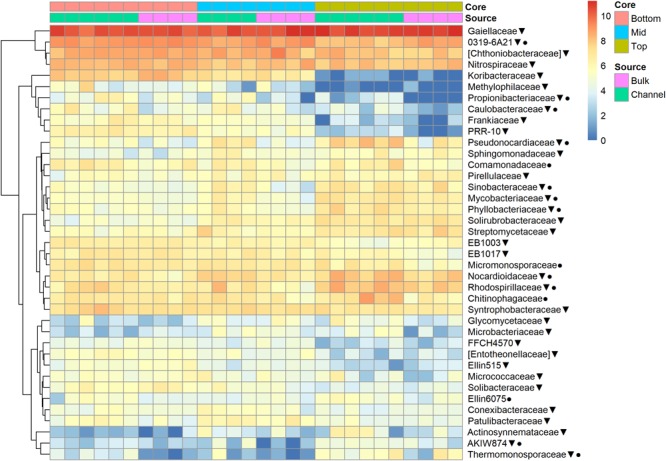
Heatmap of bacterial family abundances [Log_2_(1+x)-transformed rarefied sequence counts] in different soil cores and drilosphere samples. Top, mid, and bottom refer to slope positions. Triangles indicate families that differ significantly among slope positions (Kruskal–Wallis test, FDR-adjusted *p*-value <0.1). Circles indicate families that differ significantly between bulk versus earthworm channel soil (Kruskal–Wallis test, FDR-adjusted *p*-value <0.1).

**FIGURE 5:**
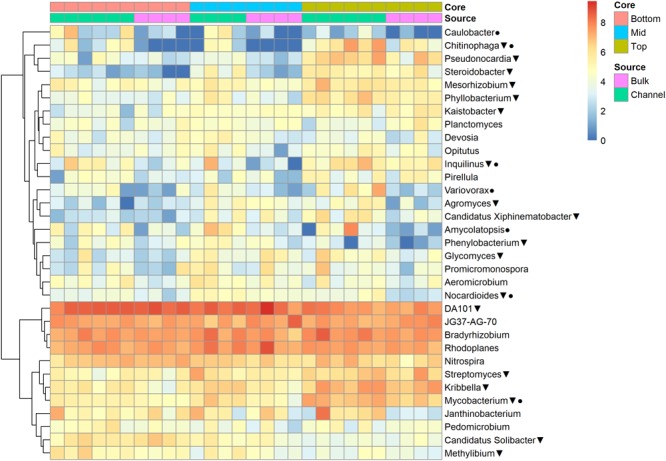
Heatmap of bacterial genera abundances [Log_2_(1+x)-transformed rarefied sequence counts] in different soil cores and drilosphere samples. Triangles indicate genera that differ significantly among slope positions (Kruskal–Wallis test, FDR-adjusted *p*-value <0.1). Circles indicate genera that differ significantly between bulk versus earthworm channel soil (Kruskal–Wallis test, FDR-adjusted *p*-value <0.1).

The relative abundances of many bacterial families differed significantly among soils from different field landscape slope positions (Figure 4). Specifically, families more prevalent in soils from the top-slope versus mid- or bottom-slope soils included Nocardioidaceae, Rhodospirillaceae, Mycobacteriaceae, Phyllobacteriaceae, Streptomycetaceae, and Solirubrobacteriaceae. In contrast, those families more abundant in mid- or bottom-slope soils than top-slope soils included Koribacteriaceae (Acidobacteria) *PRR-10*, Methylophilaceae, and Propionibacteriaceae (Figure 4). Similarly, at the genus level, *Streptomyces, Kribbella, Mycobacterium, Chitinophaga, Pseudonocardia, Phyllobacterium, Mesorhizobium*, and *Steroidobacter* were relatively more abundant in top-slope soils than mid- or bottom-slope soils. Members of DA101 (*Verrucomicrobium*) and *Candidatus* Solibacter were of greater relative abundance in mid- and bottom-slope soils than top-slope soils (Figure 5).

Despite the major role of landscape slope position in determining bacterial community structure, a small number of bacterial taxonomic groups were enriched in earthworm burrows.

**FIGURE 6:**
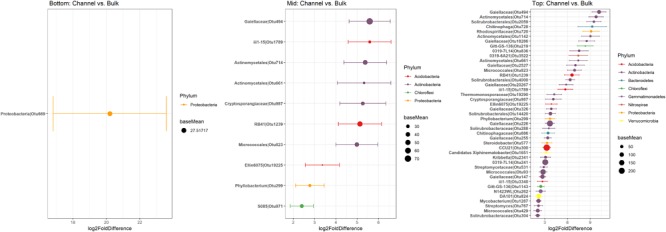
Differentially abundant OTUs between bulk and earthworm channel soil. The *x*-axis represents the DESeq2 estimated log2-fold difference in abundances between bulk and earthworm channel soil. Points are colored by the phylum to which they were classified and the size of the points indicates the mean abundance of that OTU among all samples.

Namely, the families Pseudonocardiaceae, Nocardiaceae, Propionibacteriaceae, Caulobacteraceae, Rhodospirillaceae, Thermomonosporaceae, Chitinophagaceae, and Comamonadaceae had higher relative abundance in earthworm channels than in bulk soil (KW-test, FDR-corrected *p*-values <0.1). Moreover, at the genus-level of classification, *Amycolatopsis* (KW-test, FDR *p* = 0.024), *Caulobacter* (KW-test, FDR *p* = 0.028), *Nocardioides* (KW-test, FDR *p* = 0.042), *Chitinophaga* (KW-test, FDR *p* = 0.028), and *Variovorax* (KW-test, FDR *p* = 0.025) were more prevalent in earthworm drilosphere versus bulk soil. Finally, at the OTU-level, some OTUs were significantly associated with bulk or drilosphere soil (Figure 6). Most of these OTUs belonged to the phylum Actinobacteria and Proteobacteria, including taxa related to *Micrococcus* and Gaiellaceae.

Bacterial richness and diversity varied significantly with slope position and between earthworm channels and bulk soil. In general, bacterial richness and diversity corresponded with the slope position, where soil from the bottom slope tended to have the highest richness and diversity and soil from the top slope tended to have the lowest (Table 1; ANOVA *F* = 22.8, 35.9, and 30.41 for richness, Shannon H′, and Simpsons 1/D, respectively; *p* < 0.001 in each case). Both Shannon H′ and Simpsons 1/D indices of diversity were significantly greater in earthworm drilospheres than in bulk soil, though individual contrasts were often not statistically significant between bulk versus drilosphere soil within cores (Table 1, ANOVA *F* = 13.55 and 19.13, *p* = 0.001 and 0.0002 for Shannon H′ and Simpsons 1/D, respectively).

**Table 1:** Mean (±standard deviation) of bacterial richness and diversity.

		Bacterial richness and diversity		
Landscape position	Soil source	Richness	Shannon H’	Simpsons 1/D
Bottom	Bulk	1334 ± 104a	6.12 ± 0.08a	193.17 ± 20.26a
	Channel	1310 ± 174a	6.20 ± 0.15a	232.92 ± 41.35a
Mid	Bulk	1213 ± 193ab	5.85 ± 0.21bc	138.85 ± 32.21b
	Channel	1366 ± 137a	6.12 ± 0.10ab	199.45 ± 10.80a
Top	Bulk	878 ± 50c	5.53 ± 0.11d	104.04 ± 17.29b
	Channel	1000 ± 84bc	5.76 ± 0.09cd	139.11 ± 15.21b
	**Core**	**22.8**	**35.9**	**30.41**
		**(<0.0001)**	**(<0.0001)**	**(<0.0001)**
	**Source**	2.43	**13.55**	**19.13**
		(0.13)	**(0.001)**	**(0.0002)**
	**Core × source**	1.13	1.32	0.565
		(0.341)	(0.287)	(0.577)

*Different letters within each column represent significant differences among groups. ANOVA *F*-values and *p*-values (parentheses) are presented for the effects of Core, Source, and the Core × source interaction. Values in bold indicate significance at *p* < 0.05.*

**FIGURE 7:**
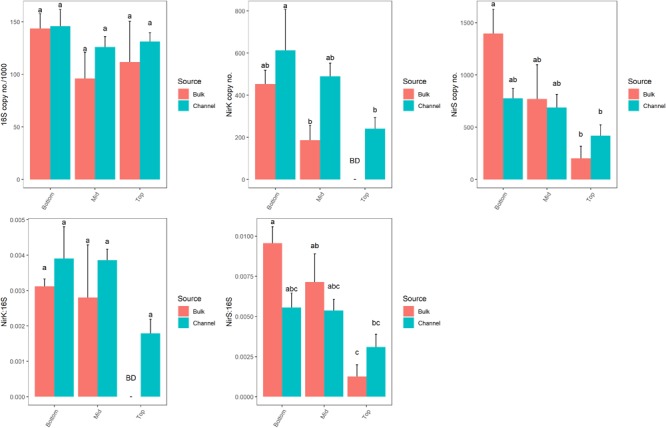
Abundances of 16S rRNA, *nirK* and *nirS* gene copy numbers from bulk soil and earthworm channels among landscape slope positions. Different letters above bars indicate statistically significant differences (Tukey’s HSD, *p* < 0.05). A statistical evaluation of differences is also presented in Supplementary Table 5.

The abundance of genes related to denitrification (*nirK* and *nirS*) varied significantly by landscape slope position (*p* = 0.043 and *p* = 0.021, respectively; Figure 7 and Supplementary Table 4). However, the numbers of bacterial 16S rRNA genes, which ranged from 9.6 × 10^4^ to 1.49 × 10^5^ copies ng^-1^ DNA, were similar across all positions (*p* = 0.097). The abundance of denitrifying genes tended to be greater in cores from bottom-slope positions compared to the top slope, although the differences were not always significant. Denitrifying genes were of very low abundance in samples taken from the top slope core, with *nirK* and *nirS* being below the detection limit in bulk soil (4 of 4 and 2 of 4 samples, respectively), and only one sample of drilosphere soil. Individually, the abundance of *nirK*, but not *nirS*, differed significantly between bulk and drilosphere soil (nested ANOVA *p* = 0.028 and *p* = 0.17, respectively); however, when comparing bulk and drilosphere samples within each core, a difference was observed between the bulk and channel soil for *nirK* at mid-slope (*t*-test, *p* = 0.027) and *nirS* at the bottom-slope (*t*-test, *p* = 0.066). Moreover, when considered together (*nirK*+*nirS*), denitrifying gene abundances were significantly greater in the drilosphere versus bulk soil (nested ANOVA, *F* = 5.47, *p* = 0.028). When compared to the abundance of the 16S rRNA gene (ratio), both denitrification genes were enriched in DNA collected from the lower two slope positions compared to the top-slope (Figure 7 and Supplementary Table 4). The ratio of *nirS*/16S rRNA genes was greater in the bulk soil at the bottom- and mid-slope compared to the top-slope position; however, soil source was not a significant factor for either gene. The *nosZ* gene was below the detectable limit (1000 copies μl^-1^ DNA extract) in all soil samples. The gene was amplified in soils from similar soil collections indicating the detection was limited by copy abundance rather than assay failure.

The relative abundances of many OTUs exhibited strong (*R* > 0.65) and significant (*p* < 0.001) correlations with absolute and relative abundances of *nirK* and *nirS* (Supplementary Figures 1, 2). These taxa represented diverse phyla, including Proteobacteria (19% of OTUs), Acidobacteria (25% of OTUs), Actinobacteria (28% of OTUs), Verrucomicrobia (9% of OTUs), and Chloroflexi (9% of OTUs), suggesting a broad diversity of bacteria were positively associated with denitrifiers. However, only one of these OTUs (OTU2688, classified to the order MND1 [beta-Proteobacteria]) varied significantly between bulk soil and earthworm channels (KW-test, *p* = 0.013). In addition to individual OTUs, bacterial richness and diversity was positively correlated with abundances of *nirK* (Sobs: *R* = 0.53, *p* = 0.006; H′: *R* = 0.68, *p* = 0.0007; 1/D: *R* = 0.72, *p* = 0.0002) and *nirS* (Sobs: *R* = 0.56, *p* = 0.008; H′: *R* = 0.49, *p* = 0.013; 1/D: *R* = 0.41, *p* = 0.041).

## Discussion

Microbial communities in soil are shaped by innumerable interactions among chemical, physical, biological, and historical variables. This work provides insight into the roles of earthworm burrowing and landscape variability, two important features in no-till cropping systems in the Palouse region, in determining soil bacterial community composition, diversity, and abundance of denitrifying populations. Bacterial community composition was related primarily to the slope position (top-, mid-, or bottom-slope), suggesting that the slope position within fields is a major determinant of within-field community variation. Many genera within the Actinobacteria were more prevalent in soils from the top-slope, including *Streptomyces, Pseudonocardia, Kribbella*, and *Mycobacterium*. In contrast, *Candidatus* Solibacter (Acidobacteria) and DA101 (Verrucomicrobia) were more prevalent in mid- and bottom-slope positions. Top-slopes in this system are highly eroded, have reduced water availability, and have lower crop yields and residue biomass compared to the bottom slopes, whereas the bottom-slopes are expected to have a deeper soil profile due to deposition of soil that eroded from higher slope positions (Fiez et al., 1995). Actinomycetes, especially *Streptomyces* and *Pseudonocardia*, are well-adapted to dry soil conditions and can survive for decades as desiccation-resistant spores (Barka et al., 2016). Moreover, these genera, as well as *Chitinophaga*, are important in disease suppression and degradation of complex organic compounds (Chung et al., 2012). A recently sequenced genome of *Candidatus* Solibacter suggests that this organism is a mixotroph that, in addition to being exceptionally adept at degrading cellulose and other plant compounds, can also use carbon monoxide as an energy source (Ward et al., 2009; Challacombe et al., 2011). Moreover, members of this genus encode genes for denitrification. Very little information exists on the biology of the genus DA101; however, the sole cultured representative of its family Chthoniobacteraceae (*Chthoniobacter flavus*) encodes *nirK* (Coyotzi et al., 2017) and is involved in the breakdown of organic substrates (Kant et al., 2011). Together, these results suggest that landscape variation in bacterial community composition, driven by within-field gradients in soil moisture, plant productivity, and soil chemistry due to landscape topology, contribute to differences in key microbial community functions such as carbon and nutrient cycling, denitrification, and plant disease suppression. However, due to sampling limitations we were unable to obtain detailed information on soil characteristics. Thus, the impact of slope position and soil edaphic factors on soil communities were not the primary focus of this work and should be explored in greater detail in future studies.

Although slope position appeared to be the primary driver of bacterial community composition in this study, a small number of bacterial groups were significantly enriched in the earthworm drilosphere. These taxa included the families AKIW874 (Acidomicrobia) and Comamonadaceae (beta-Proteobacteria), and members of the genera *Variovorax* (family Comamonadaceae) and *Amycolatopsis*. *Variovorax* and *Amycolatopsis* are both common soil bacteria. *Variovorax* is a possible endosymbiont of nephridia of some earthworm species and is frequently a part of earthworm gut microflora (Davidson et al., 2013). *Variovorax* is often involved in degradation of xenobiotic compounds, can act as a plant growth promoter (Pereira et al., 2016), and has previously been shown to be enriched in soil amended with earthworms (Cao et al., 2015). *Amycolatopsis* species are frequent producers of geosmin, a volatile natural product responsible for the “earthy” smell of soil, which is hypothesized to attract earthworms and springtails to act as dispersal vectors as bacterial spores adhere to exoskeletons or epidermis and are transported with the animal (Hopwood, 2007; Zhao et al., 2010). Dispersal and deposition of antibiotic-producing actinomycetes, such as *Amycolatopsis*, may also influence the breakdown of plant residues via inhibition of more efficient decomposer fungi (van der Meij et al., 2017). Finally, since both *Variovorax* and *Amycolatopsis* also associate with plant roots, enrichment of these taxa in the drilosphere may subsequently increase plant root colonization and benefit plant health and productivity via growth promotion, protection from disease or xenobiotics, and enhanced nutrient cycling.

Most of the individual bacterial taxa enriched in drilosphere versus bulk soil were members of Actinobacteria, including Micrococcales, Gaiellaceae, Solirubrobacterales, and *Mycobacterium*. However, these taxa could not be classified to finer taxonomic levels and may represent uncultured or understudied species. Very little is known about the members of *Gaiella* or Solirubrobacterales, which were recently proposed as separate families within the Class Thermoleophilia (Albuquerque et al., 2011). Members of the Micrococcales may be especially well-adapted to growth in low moisture environments, perhaps due to their ability to produce biofilms. Bacterial biofilm residues may contribute to the stability of earthworm channels in soil. *Mycobacterium* species, including potentially pathogenic *M. tuberculosis*, has been demonstrated to be efficiently dispersed in soil via earthworm activity (Fischer et al., 2003). Together, enrichment of many poorly characterized taxa in the drilosphere suggest that many bacterial groups have unidentified direct associations with earthworms or effectively colonize burrow channels differently from the bulk soil. However, because the difference between drilosphere and bulk soil communities was strongest in top-slope positions (accounting for 39% of variation in community composition), intermediate in mid-slope positions (accounting for 22% of variation in community composition), and weakest in bottom-slope positions (accounting for 14% of variation in community composition), the impact of earthworm burrows on specific OTUs also depended on slope position. The top-slope position had the most rapid saturated hydraulic conductivity, suggesting these soils were more porous, and may reflect a more extensive network of deeper worm channels. Thus, the influence of earthworm burrowing activity on microbial community structure in the drilosphere appears to depend on landscape and slope characteristics. This variation may be due to differences in earthworm activity across the landscape, different ages of earthworm channels, or differences in soil properties that structure microbial communities in bulk soil.

In addition to differences in bacterial community composition between drilosphere and bulk soil, earthworm burrows also harbored a greater diversity of bacteria. The consistently higher bacterial diversity within burrows is likely due to the greater availability of C and N in earthworm burrows than non-impacted (or bulk) soil (Tiunov and Scheu, 1999). Specifically, earthworms provide crucial resources for bacterial activity by pulling large amounts of organic residues below ground, leaving mucilage and casts within burrows, and thus may contribute to the maintenance of microbial diversity in burrow linings. Moreover, earthworm activity or increased water infiltration rates may generate higher rates of dispersal of microbial populations among soil strata within earthworm burrows and contribute to the observed differences in bacterial diversity.

Similar to bacterial community composition, the abundance of denitrification genes (*nirK* and *nirS*) varied primarily among slope positions, where they were more abundant in bottom-slope position and least abundant in top-slope position. This difference in the gene distribution at the 50 cm depth core is consistent with previous work by Shrewsbury et al. (2016) at the same site (CAF), which revealed that in the 0–5 cm depth there were differences in the abundance of denitrifying genes between upper and lower slope positions, and that denitrifying gene abundances were related to total soil C. However, Shrewsbury et al. (2016) also found that variation in denitrifier gene abundances across the landscape were significant only in winter.

Despite potential seasonal variation in denitrifier gene abundances, *nirK* tended to be more abundant in earthworm burrows, suggesting that the drilosphere is a hotspot of bacterial denitrification in soil. Alternatively, because denitrifying bacteria are a key part of the earthworm gut microflora (Depkat-Jakob et al., 2010), the high abundance of *nirK* in burrows may reflect deposition of earthworm gut inhabitants. The bacterial genera *Bradyrhizobium, Rhodoplanes, Streptomyces*, and *Janthinobacterium* include denitrifiers (Shapleigh, 2013). *Janthinobacterium* was the only genera that was enriched in the burrow compared to bulk soil and only in the top core. The members of the families Solibacteraceae, Streptomycetaceae, Comamonadaceae, Koribacteraceae, and Propionibaceteriaceae were recently described as active denitrifiers in an agricultural soil (Coyotzi et al., 2017). Indeed, correlations among OTU abundances and abundances of denitrifying genes suggest that the denitrifiers in burrow linings in the deep drilosphere are primarily associated with the groups Actinobacteria, Acidobacteria, and Verrucomicrobia.

Taken together, the results of this study provide valuable insights into the roles that anecic earthworms play in structuring soil bacterial communities at different landscape positions. Bacterial communities were influenced by both slope position and earthworm activity. Increased bacterial diversity, as well as enrichment for denitrifiers and taxa related to nutrient cycling and plant health in the drilosphere suggests that increases in the densities and activities of anecic earthworms in no-till systems will impact nutrient dynamics and plant productivity via modification of the soil microbiome.

## Author Contributions

DS, TP, and JJ-M designed the study. EB, KK, JN, and DH provided the soil cores for the study. DS conducted the sequencing and microbial community analysis. CR conducted the qPCR of bacterial genes. DS and TP wrote the initial draft of the manuscript. JJ-M, CR, EB, KK, JN, and DH contributed to revising the manuscript.

## Conflict of Interest Statement

The authors declare that the research was conducted in the absence of any commercial or financial relationships that could be construed as a potential conflict of interest.
