# Supplementary material for: Mining the Drilosphere: Bacterial Communities and Denitrifier Abundance in a No-Till Wheat Cropping System
Source: Front Microbiol. 2019 Jun 26;10:1339. doi: 10.3389/fmicb.2019.01339 (PMC6611406; doi:10.3389/fmicb.2019.01339)
Supplement: Supplementary file 1 [file Table_1.DOCX]

Supplemental Table 1. Classification of soil horizons conducted by USDA-ARS scientists on site at each of the soil core locations.

| Core Location | Soil Type | Elevation | Horizons | Depth (cm) |
| --- | --- | --- | --- | --- |
| Top Core | Palouse | 811.6 | Ap | 19 |
|  |  |  | BA | 48 |
|  |  |  | Bw1 | 82 |
|  |  |  | Bw2 | 122 |
|  |  |  | Bwb | 153 |
|  |  |  |  |  |
| Middle Core | Palouse | 801.4 | Ap | 23 |
|  |  |  | A | 38 |
|  |  |  | BA | 60 |
|  |  |  | BW | 99 |
|  |  |  | Bwb | 153 |
|  |  |  |  |  |
|  |  |  |  |  |
| Bottom Core | Thatuna | 791.6 | Ap | 16 |
|  |  |  | A1 | 21 |
|  |  |  | A2 | 60 |
|  |  |  | AB | 88 |
|  |  |  | E/B | 125 |
|  |  |  | Bwb | 153 |
