# Supplementary material for: Mining the Drilosphere: Bacterial Communities and Denitrifier Abundance in a No-Till Wheat Cropping System
Source: Front Microbiol. 2019 Jun 26;10:1339. doi: 10.3389/fmicb.2019.01339 (PMC6611406; doi:10.3389/fmicb.2019.01339)
Supplement: Supplementary file 2 [file Table_2.docx]

Supplemental Table 2. Bulk density measurements with depth determined from replicate soil cores extracted from the side wall of each pit.

|  |  | Bulk Density (g/cm^3^) | | |
| --- | --- | --- | --- | --- |
| Core Location | Depth (cm) | Sample 1 | Sample 2 | Average |
| Top-slope | 0 – 5 | 1.12 | 1.10 | 1.11 |
| Top-slope | 20 – 25 | 1.35 | 1.44 | 1.40 |
| Top-slope | 35 – 40 | 1.37 | 1.37 | 1.37 |
|  |  |  |  |  |
| Mid-slope | 0 – 5 | 1.18 | 1.21 | 1.20 |
| Mid-slope | 20 - 25 | 1.15 | 1.27 | 1.21 |
| Mid-slope | 35 - 40 | 1.26 | 1.32 | 1.29 |
|  |  |  |  |  |
| Bottom-slope | 0 – 5 | 1.08 | 1.17 | 1.13 |
| Bottom-slope | 20 - 25 | 1.40 | 1.26 | 1.33 |
| Bottom-slope | 35 - 40 | 1.28 | 1.35 | 1.32 |
