# Supplementary material for: Mining the Drilosphere: Bacterial Communities and Denitrifier Abundance in a No-Till Wheat Cropping System
Source: Front Microbiol. 2019 Jun 26;10:1339. doi: 10.3389/fmicb.2019.01339 (PMC6611406; doi:10.3389/fmicb.2019.01339)
Supplement: Supplementary file 3 [file Table_3.docx]

Supplemental Table 3. Primers and thermocycling conditions

| Target | Primer*^a^* | Sequence (5’-3’) | Reference^b^ | Standard curve efficiency, R^2^, and range of copies μL^-1^ |
| --- | --- | --- | --- | --- |
| Bacterial 16S, V3 region | | |  | >92%, 0.996, 1.9e^7^-1.9e^3^ |
|  | 338F | ACTCCTACGGGAGGCAGCAG | 1 |  |
|  | 518R | ATTACCGCGGCTGCTGG | 1 |  |
| *nirK* | | |  | >92%, >0.994, 1.6e^6^-1.6e^2^ |
|  | nirK876 | ATYGGCGGVCAYGGCGA | 2,3 |  |
|  | nirK1040 | GCCTCGATCAGTTTRTGGTT | 2 |  |
| *nirS* | | |  | >95%, 0.996, 4.0e^6^ - 4.0e^1^ |
|  | nirS2F | TACCACCCSGARCCGCGCGT | 4 |  |
|  | nirS3R | GCCGCCGTCRTGVAGGAA | 4 |  |
| *nosZ* | | |  | >90%, 0.998, 1.0e^6^ – 50 |
|  | nosZ1F | WCSYTGTTCMTCGACAGCCAG | 5 |  |
|  | nosZ1R | ATGTCGATCARCTGVKCRTTYTC | 5 |  |

*^a^* Primer concentrations were 0.4 μM except for 338F/518R which were 0.2 μM.

^b^. References

1. Øvreås L, Forney L, Daae F, Torsvik V (1997) Distribution of bacterioplankton in meromictic Lake Saelenvannet, as determined by denaturing gradient gel electrophoresis of PCR-amplified gene fragments coding for 16S rRNA. Appl Environ Microbiol 63:3367-3373

2. Henry S, Baudoin E, López-Gutiérrez JC, Martin-Laurent F, Brauman A, Philippot L (2004) Quantification of denitrifying bacteria in soils by *nirK* gene targeted real-time PCR. J Microbiol Methods 59:327-335

3. Henry S, Baudoin E, López-Gutiérrez JC, Martin-Laurent F, Brauman A, Philippot L (2005) Corrigendum to "Quantification of denitrifying bacteria in soils by *nirK* gene targeted real-time PCR" [J. Microbiol. Methods 59 (2004) 327-335]. J Microbiol Methods 61:289-290

4. Braker G, Fesefeldt A, Witzel K-P (1998) Development of PCR primer systems for amplification of nitrite reductase genes (*nirK* and *nirS*) to detect denitrifying bacteria in environmental samples. Appl Environ Microbiol 64:3769-3775

5. Henry S, Bru D, Stres B, Hallet S, Philippot L (2006) Quantitative detection of the *nosz* gene, encoding nitrous oxide reductase, and comparison of the abundances of 16S rRNA, *narG*, *nirK*, and *nosZ* genes in soils. Appl Environ Microbiol 72:5181-5189
