# Supplementary material for: Mining the Drilosphere: Bacterial Communities and Denitrifier Abundance in a No-Till Wheat Cropping System
Source: Front Microbiol. 2019 Jun 26;10:1339. doi: 10.3389/fmicb.2019.01339 (PMC6611406; doi:10.3389/fmicb.2019.01339)
Supplement: Supplementary file 4 [file Table_4.docx]

Supplemental Table 4: Mean (± standard deviation) of gene abundances. Different letters within each column represent significant differences among groups. ANOVA F-values and p-values (parentheses) are presented for the effects of Core, Source, and the Core × Source interaction. Values in bold indicate significance at *p*<0.05.

|  |  | **Gene abundance (copies ng^-1^ DNA) and enrichment (ratio)** | | | |
| --- | --- | --- | --- | --- | --- |
| **Landscape Position** | **Soil Source** | ***nirK*** | ***nirS*** | ***nirK/16S*** | ***nirS/16S*** |
| Bottom | Bulk | 453±131ab | 1395±460a | 0.0031±0.0004a | 0.0095±0.0021a |
|  | Channel | 735±412a | 775±238ab | 0.0047±0.0012a | 0.0056±0.0022abc |
| Mid | Bulk | 248±83b | 769±656ab | 0.0037±0.0028a | 0.0071±0.0035ab |
|  | Channel | 469±128ab | 671±204ab | 0.0040±0.0008a | 0.0053±0.0011abc |
| Top | Bulk | BD | 403±18b | BD | 0.0025±0.0002c |
|  | Channel | 290±50b | 502±169b | 0.0022±0.0005a | 0.0037±0.0014bc |
|  | **Core** | **3.85** | **4.79** | **7.22** | **13.33** |
|  |  | **(0.043)** | **(0.021)** | **(0.0039)** | **(0.0002)** |
|  | **Source** | **5.5** | 2.96 | 3.9 | 2.36 |
|  |  | **(0.032)** | (0.102) | (0.061) | (0.139) |
|  | **Core × Source** | 0.032 | 2.112 | 0.25 | **4.36** |
|  |  | (0.86) | (0.149) | (0.78) | **(0.0255)** |
