# Supplementary material for: Mining the Drilosphere: Bacterial Communities and Denitrifier Abundance in a No-Till Wheat Cropping System
Source: Front Microbiol. 2019 Jun 26;10:1339. doi: 10.3389/fmicb.2019.01339 (PMC6611406; doi:10.3389/fmicb.2019.01339)
Supplement: Supplementary file 5 [file Table_5.docx]

**Supplemental Methods**

Thermocycling protocol for qPCR assays.

Thermocycling was performed with a StepOnePlus Real-Time PCR System (Life Technologies) using a 2- or 3-step amplification protocol. Following an initial 15 min activation step, bacterial 16S rRNA and *nirK* genes were amplified with 40 cycles of 95 °C for 15 s and 60 °C for 1 min. The *nirS* gene was amplified in a 3-step reaction with an initial activation of 95 °C for 15 min followed by 40 cycles of 95 °C for 15 s, 63 °C for 1 min, and an extension of 72 °C for 30 s. A touchdown protocol was used to amplify the *nosZ* gene with a 95 °C for 15 min activation step, 6 cycles of 95 °C for 15 s, 1 degree touch down from 65 °C to 60 °C for 1 min and 80 °C for 30 min, followed by 40 amplification cycles of 95 °C for 15 s, 60 °C for 1 min, and 80 °C for 30 s.

MiSeq Data Processing

Forward and reverse Illumina reads were paired using PEAR (v0.9.6) (Zhang et al., 2014). Barcodes and primer sequences were removed with cutadapt (v1.91) (Martin, 2011), and sequences with ambiguous bases or shorter than 350 bp were removed. Processed sequences were clustered following the UPARSE pipeline (Edgar, 2013) using vsearch (Rognes et al., 2016) for all steps with the exception of OTU clustering which used usearch (v8.1) (Edgar, 2013). Briefly, reads were quality filtered using a maximum expected error rate of 1, dereplicated, and singletons removed prior to OTU clustering at 97% similarity threshold using the cluster_otus command. Processed reads were then mapped to OTU clusters to generate an OTU abundance table.

Edgar, R. C. (2013). UPARSE: highly accurate OUT sequences from microbial amplicon reads. *Nat. Methods* 10, 996-998.

Martin, M. (2011). Cutadapt removes adapter sequences from high-throughput sequencing reads. *EMBnet J.* 17:10.

Rognes, T., Flouri, T., Nichols, B., Quince, C., and Mahé, F. (2016). VSEARCH: a versatile open source tool for metagenomics. *PeerJ* 4:e2584.

Zhang, J., Kobert, K., Flouri, T., and Stamatakis, A. (2014). PEAR: a fast and accurate illumine paired end read merger. *Bioinformatics* 30, 614-620.
