# Supplementary figures and images for: Mining the Drilosphere: Bacterial Communities and Denitrifier Abundance in a No-Till Wheat Cropping System
Source: Front Microbiol. 2019 Jun 26;10:1339. doi: 10.3389/fmicb.2019.01339 (PMC6611406; doi:10.3389/fmicb.2019.01339)

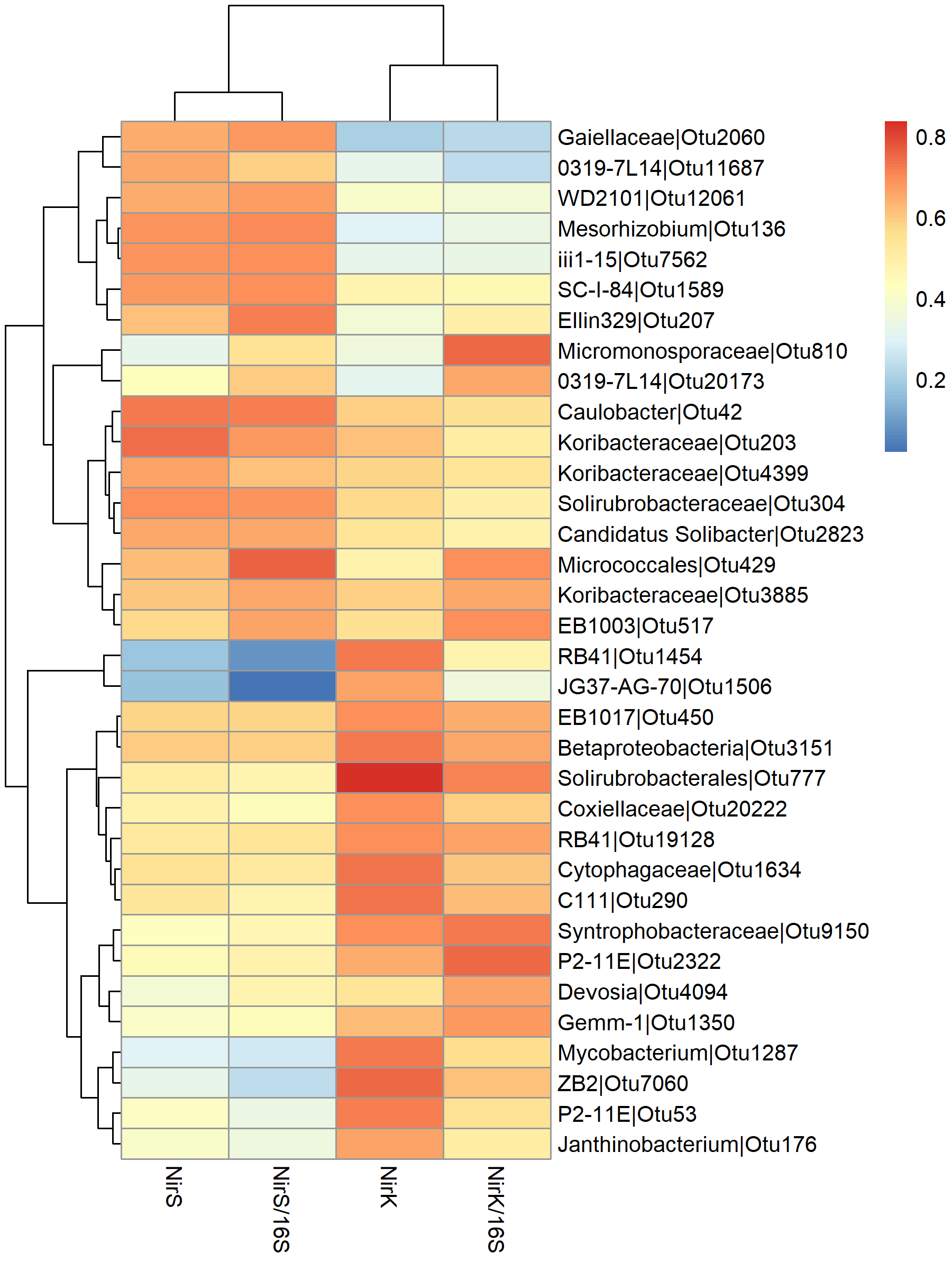

Supplement: Supplementary file 6 [file Image_1.TIFF]

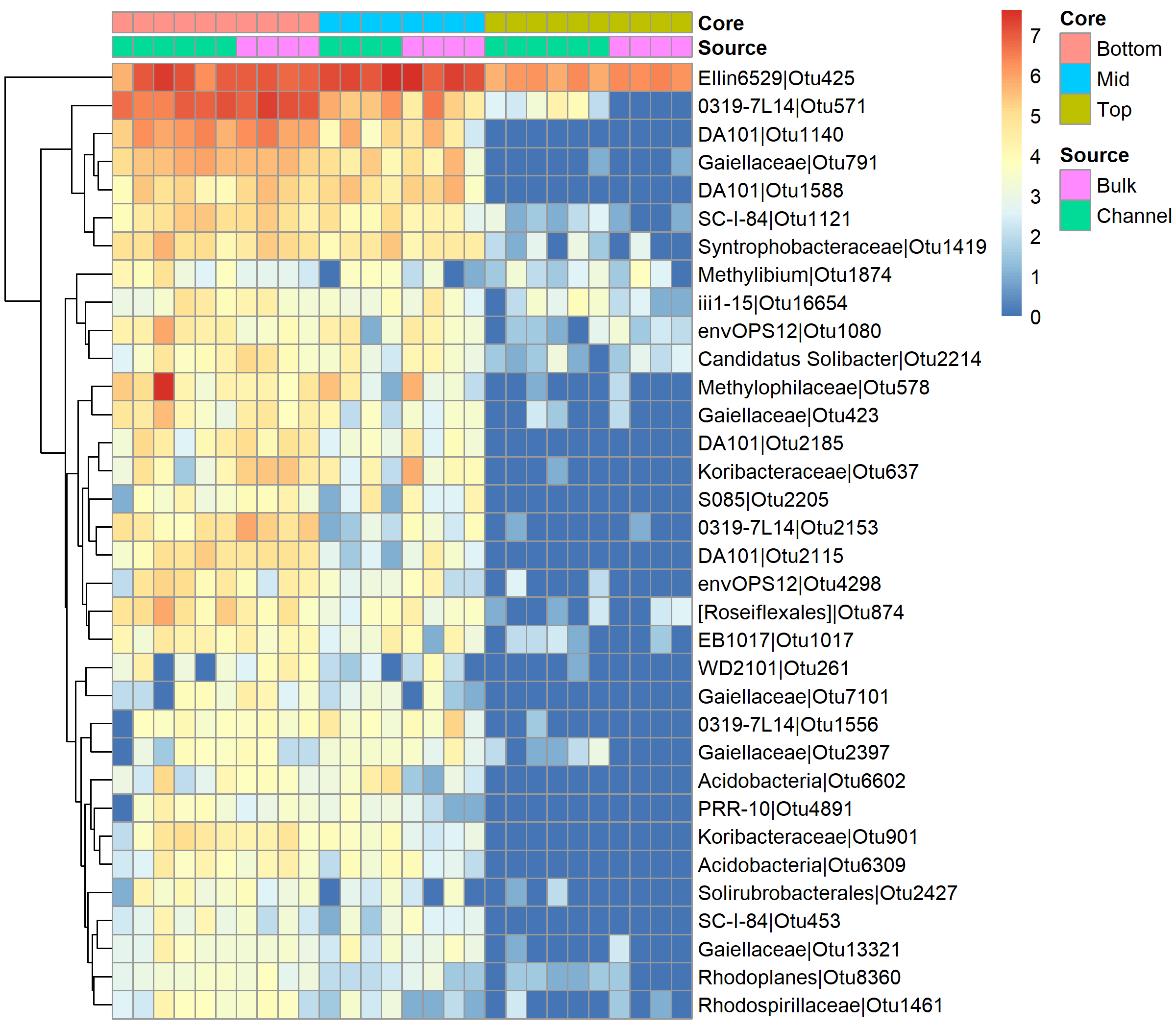

Supplement: Supplementary file 7 [file Image_2.TIFF]
